# DNA damage in B and T lymphocytes of farmers during one pesticide spraying season

**DOI:** 10.1007/s00420-015-1024-3

**Published:** 2015-02-03

**Authors:** Pierre Lebailly, Gladys Mirey, Fabrice Herin, Yannick Lecluse, Bernard Salles, Elisa Boutet-Robinet

**Affiliations:** 1 Univ. Caen Basse-Normandie, Cancers et Préventions, IFR146 ICORE, 14000 Caen, France; 2 INSERM, UMR 1086, 14000 Caen, France; 3 Centre de Lutte Contre le Cancer François Baclesse, 14076 Caen, France; 4 INRA, UMR 1331, Toxalim, Research Centre in Food Toxicology, 31027 Toulouse, France; 5 Université de Toulouse, UPS, UMR 1331, Toxalim, 31062 Toulouse, France; 6 INSERM, UMR 1027, Université de Toulouse, UPS, 31000 Toulouse, France; 7 CHU Toulouse, Service des Maladies Professionnelles et Environnementales, 31059 Toulouse, France; 8 Equipe « Génotoxicité et Signalisation » - Toxalim - UMR 1331, INRA/INP/UPS, 180 chemin de Tournefeuille, BP 93173, 31027 Toulouse Cedex 3, France

**Keywords:** Comet assay, DNA damage, Lymphocytes, Occupational exposure, Pesticides

## Abstract

**Purpose:**

The effect of one pesticide spraying season on DNA damage was measured on B and T lymphocytes among open-field farmers and controls.

**Methods:**

At least two peripheral blood samples were collected from each individual: one in a period without any pesticide application, several weeks after the last use (January, at period P0), and another in the intensive pesticide spraying period (May or June, at period P4). DNA damage was studied by alkaline comet assay on isolated B or T lymphocytes.

**Results:**

Longitudinal comparison of DNA damage observed at both P0 and P4 periods revealed a statistically significant genotoxic effect of the pesticide spraying season in both B (*P* = 0.02) and T lymphocytes (*P* = 0.02) in exposed farmers. In contrast, non-farmers did not show any significant modifications. DNA damage levels in B and T lymphocytes were significantly higher in farmers than in non-farmers during the P4 period (*P* = 0.003 and *P* = 0.001 for B and T lymphocytes, respectively) but not during the P0 period. The seasonal effect observed among farmers was not correlated with either total farm area, farm area devoted to crops or recent solar exposure. On average, farmers used pesticides for 21 days between P0 and P4. Between the two time points studied, there was a tendency for a potential effect of the number of days of fungicide treatments (*r* ^2^ = 0.43; *P* = 0.11) on T lymphocyte DNA damage.

**Conclusions:**

A genotoxic effect was found in lymphocytes of farmers exposed to pesticides, suggesting in particular the possible implication of fungicides.

**Electronic supplementary material:**

The online version of this article (doi:10.1007/s00420-015-1024-3) contains supplementary material, which is available to authorized users.

## Introduction

Pesticides are a heterogeneous group of chemicals used worldwide. They include insecticides, herbicides, fungicides and disinfectants, and are used by farmers to protect their crops and improve agricultural production. Epidemiologic studies have reported a lower incidence of overall cancer among farmers. However, a higher incidence of certain types of cancers, particularly hematopoietic cancers (Merhi et al. 2007; Van Maele-Fabry et al. 2007), soft tissue sarcoma (Kogevinas et al. 1995), lung cancer (Veglia et al. 2007) and prostate cancer (Parent et al. 2009; Van Maele-Fabry and Willems 2004), has been described (for review Alavanja and Bonner 2012). There are several etiologic clues to farming-related pathologies such as ultraviolet light, zoonotic viruses and pesticides, all being clearly or potentially genotoxic. Genotoxicological biomonitoring in human samples is a useful approach to evaluate pesticide genotoxicity. Genotoxic exposure may induce either directly or indirectly DNA single- and/or double-strand breaks that could induce chromosomal alterations as revealed by cytogenetic methods such as karyotyping, micronuclei assays or sister chromatid exchange analysis. The single-cell gel electrophoresis assay, also named the comet assay, has been widely used under alkaline conditions to monitor the occurrence of strand breaks and alkali-labile sites. Since the comet assay is a useful tool in human biomonitoring studies (Faust et al. 2004; Moller et al. 2000), we used it on peripheral blood lymphocytes (PBL) from farmers in order to assess their level of DNA damage.

In biomonitoring studies using the alkaline comet assay, it has been suggested that B lymphocytes are more sensitive to occupational exposure to benzene or polycyclic aromatic hydrocarbons (Sul et al. 2002, 2003). These studies suggest that B lymphocytes are useful targets since they are more sensitive than the total lymphocyte population. In addition, in vitro experiments have demonstrated differential responses to various genotoxic agents in B and T lymphocytes, and it is known that the nature and the doses of genotoxic agents used can account for the difference in sensitivity (for review see Weng and Morimoto 2009). For example, B lymphocytes displayed a higher sensitivity than T lymphocytes in vitro with low doses of gamma radiation (<1 Grays). On the other hand, T lymphocytes were more sensitive than B lymphocytes at higher doses (>2 Grays) of gamma radiation (Vral et al. 2001). The reasons for these differential responses to genotoxic agents in lymphocyte subpopulations have not been totally elucidated and could involve (1) the life span differences of these lymphocyte subpopulations or (2) the higher capacity of DNA repair synthesis for T cells compared to B cells (Koistinen and Vilpo 1986).

A previous study demonstrated an increase in DNA adduct level among farmers during the spraying season (Le Goff et al. 2005). Using the same blood samples, the present study using comet assays on PBL examines whether pesticide exposure at different times during one spraying season increases DNA damage. Only a few studies have investigated lymphocytes collected at different times before and after pesticide exposure (Bull et al. 2006). Indeed, the majority of studies were cross-sectional, comparing one sample per individual between an exposed and a control group. Longitudinal studies involving repeated sampling from the same subject may avoid the influence of some confounding factors like age, diet, exercise, gender and smoking (Moller et al. 2000). Therefore, we decided to test samples collected at different times during one spraying period. In addition, we isolated B and T lymphocytes in order to compare DNA damage in these two cell subpopulations. Our results show the effect of a pesticide spraying season on both T and B lymphocytes.

## Materials and methods

### Populations and sampling chronology

Twenty-six (20 with cryopreserved viable lymphocytes) French open-field farm owners (FO) occupationally exposed to various pesticides, and 29 non-farmers (22 with viable cryopreserved lymphocytes) who were hospital workers living in a rural area (non-exposed group) were included in this study (Le Goff et al. 2005). After the study was approved by the local ethical committee, informed consent was obtained from each individual prior to the beginning of the study. General characteristics of both groups are shown in Table 1, while details regarding farms, crops and pesticide use are given in Table 2.

**Table 1:** Population characteristics (all males)

	Non-exposed group	Farm owners (exposed group)
Number of volunteers	22	20
Age (years)		
Mean ± SD (min–max)	39 ± 10 (25–52)	38 ± 7 (27–51)
Smoking habits		
Smokers	3 (14 %)	5 (25 %)
Non-smokers	18 (82 %)	15 (75 %)
Missing	1 (4 %)	0 (0 %)
Daily alcohol consumption	Not collected	
No		5 (25 %)
Yes		12 (60 %)^a^
Missing		3 (15 %)

^a^For these individuals, the mean daily alcohol consumption was 34 ± 23 (mean ± SD in g per day)

**Table 2:** Crop areas and pesticide exposure characteristics

	Characteristics of crop areas and pesticide exposure in FO (*n* = 20)
Total farm area (ha) mean ± SD (min–max)	137 ± 62 (58–307)
Area devoted to crops (ha) mean ± SD (min–max)	123 ± 50 (55–230)
Wheat (%)^a^	20/20 (100 %)
Wheat area (ha)^b^ mean ± SD (min–max)	62 ± 28 (30–128)
Peas (%)^a^	19/20 (95 %)
Peas area (ha)^b^ mean ± SD (min–max)	23 ± 14 (6–57)
Corn (%)^a^	10/20 (50 %)
Corn area (ha)^b^ mean ± SD (min–max)	22 ± 23 (5–75)
Beet (%)^a^	15/20 (75 %)
Beet area (ha)^b^ mean ± SD (min–max)	10 ± 6 (4–24)
Fruit growing (%)^a^	5/19 (1 missing data) (25 %)
Fruit growing (ha)^b^ mean ± SD (min–max)	4 ± 3 (1–8)
Truck farming (%)^a^	3/20 (15 %)
Truck farming (ha)^b^ mean ± SD (min–max)	17 ± 17 (1–36)
Duration of pesticide use (years) mean ± SD (min–max)	17 ± 8 (4–31)
Pesticide treatments (number of days of treatment between S0 and S4) for *n* = 15 (5 missing data)	
Herbicide treatments mean ± SD (min–max)	12 ± 8 (2–33)
Insecticide treatments mean ± SD (min–max)	3 ± 3 (0–13)
Fungicide treatments mean ± SD (min–max)	6 ± 5 (0–18)

^a^Number of FO who grow this crop

^b^Restricted to FO who grow this crop

All individuals in both groups were male, under 52 years old and had an occupational activity. There was no statistically significant difference in age between the groups (*P* = 0.73, the mean age was 39 years in the non-exposed group and 38 years in the FO group). A minority of subjects was current smokers with no significant differences (14 % of people in the non-exposed group and 25 % in the FO group; *χ* ^2^, *P* = 0.39) (Table 1). Table 2 summarizes the pesticide exposure characteristics. On average, 90 % of the total farm area was devoted to crops and the average crop area was 123 ha (ranging from 55 to 230 ha). The crop areas were located in the *Département du Calvados* (Normandy, France) and dedicated mainly to open-field crops for the production of wheat, corn and peas (see Table 2 for details). Most of the FO (17/20) had grown four or five different types of crops.

In 1997 (FO) and 1999 (non-exposed group), two blood samples per individual were collected: One was taken in a period without any pesticide application, several weeks after the last use (January, S0 sample in period P0), and one was collected in the intensive pesticide spraying period (May or June, S4 sample in period P4). In the FO group, another sample was collected after the first day and/or after the second day of pesticide spraying (sample S2 and S3 in periods P2 and P3, collected in 13 and 3 subjects, respectively).

### Processing of samples and comet assays

Mononuclear cells were Ficoll separated from whole blood and cryopreserved in liquid nitrogen as previously described (Lebailly et al. 1998a). These conditions of preparation and conservation are suitable for long-term conservation and do not influence comet assay results (Dusinska and Collins 2008). Mononuclear cells from the whole blood sample of one volunteer from the *Etablissement Français du Sang* (EFS) were isolated with the same procedure and aliquoted prior to cryopreservation to serve as an internal standard for further experiments. B or T lymphocytes were negatively selected using magnetic beads (Dynabeads^®^ Untouched^®^ Human B or T cells Kits, Invitrogen) and a DynaMag^®^ magnet, according to the supplier’s recommendations, with minor modifications (all the steps in the protocol were performed at 4 °C, without any agitation, tilting or rotation). Immediately after the B or T lymphocytes were isolated, the comet assay was performed exactly as previously described (Lebailly et al. 2003). An aliquot of the same EFS volunteer (internal standard) was defrosted and used in each experiment. After staining with 50 µL of ethidium bromide (2 µg/mL), slides were observed at 20× magnification using a Nikon 50i fluorescence microscope. Images were analyzed with a Luca S camera and the Komet 6 software (Kinetic Imaging). Fifty cells per slide and two slides per sample were analyzed. Experimenters were blinded to the status of subjects (farm owners or non-exposed) from whom samples came. The median olive tail moment (OTM) (which is the product of the percentage of DNA in the tail and the distance between the center of the head and the barycenter of the tail as defined by Olive et al. 1990) was calculated from these 100 values. The OTM parameter was chosen as it is a widely used parameter in comet assays (Kumaravel et al. 2009). Cell viability was evaluated by the trypan blue exclusion method. The mean viability of the internal standard was 95 % ± 3 (mean ± SD). There was no statistically significant correlation between the viability and the median OTM of the standard. Viability of samples after thawing was significantly higher in samples from FO (95 % ± 3) than in those from the non-exposed group (92 % ± 4) in the P0 period (Student’s *t* test, *P* = 0.01). In the P4 period, the viability of samples from FO was also higher than in those from the non-exposed group (95 % ± 4 vs. 90 % ± 4, *P* = 0.001). There was no correlation between DNA damage and lymphocyte viability, suggesting that the observed genotoxicity was unrelated to any cytotoxic effect.

### Intra- and inter-experimental variability

Several steps in the comet assay protocol may affect both intra- and inter-assay variability: slide preparation, cell lysis, electrophoresis conditions and variations in comet cell analysis. To take this variability into account, 100 cells of each sample were analyzed from two independent slides examined in the same experiment. To evaluate the inter-experimental variability, two slides containing internal standard cells were included in each experiment, as suggested by others (De Boeck et al. 2000; Holz et al. 1995; Moller et al. 2010).

### Statistical analysis

When samples were tested in the same experiment (i.e., in the same electrophoretic run), median OTM values were used for statistical analysis. If samples were not tested in the same experiment, OTM values were normalized by the standard OTM value before statistical analysis. For categorical variables, the *χ* ^2^ or Fisher’s exact tests were used when appropriate. For continuous variables, the Student’s *t* test for paired samples, the Wilcoxon nonparametric test, a linear regression model or the Spearman correlation test were used when appropriate. A two-sided *α* level of 0.05 was used. Statistical analyses were performed with the software Stata release 11.

## Results

### Intra- and inter-experimental variability

**Fig. 1:**
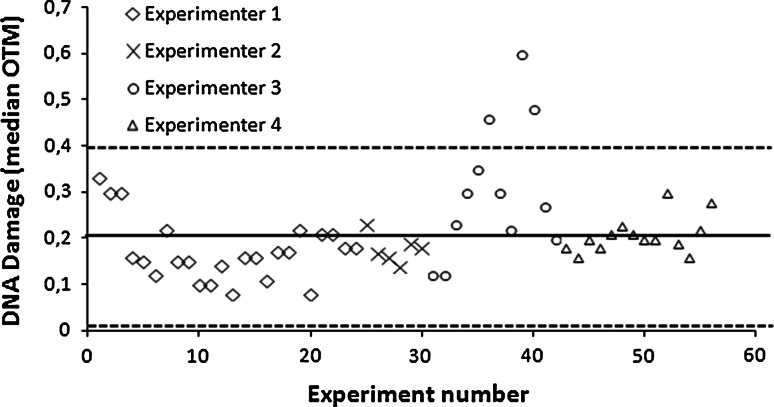
Variability of the internal standard observed in 56 independent experiments. Experiments were performed from May 2009 to September 2009 by experimenter 1; from January 2010 to February 2010 by experimenter 2; from March 2010 to May 2010 by experimenter 3; and from April 2011 to July 2011 by experimenter 4. One experiment is defined as one electrophoretic run. The two *dotted horizontal lines* represent the range of mean ± 2 SD, representing an acceptable variability for the comet assay, as previously described (De Boeck et al. 2000). The *solid horizontal line in the middle* of this range represents the mean of the data. Statistical analyses performed for comparisons of DNA damage levels between non-exposed individuals and farm owners were conducted with and without exclusion of the three experiments, for which the OTM of the standard was higher than this range

Figure 1 shows the results of the 56 independent alkaline comet experiments on aliquots from the same EFS volunteer (expressed as median olive tail moment, OTM) performed by four independent experimenters. Since the relative standard deviation of these results was 46 %, we normalized the results in order to compare samples from different experiments. To do so, we divided the median OTM of each sample by the median OTM of the internal standard obtained from the sample of an EFS volunteer that had been aliquoted and frozen. The results thus obtained were termed “relative OTMs.” This OTM parameter was then used for cross-sectional analysis in order to minimize the influence of inter-experiment variability, as results compared in such analysis were obtained from independent experiments. Most of the results (except 3 of the 56 experiments) were in the range of the mean ± 2 SD (corresponding of a median OTM between 0.01 and 0.4), representing an acceptable variability for the comet assay, as previously described (De Boeck et al. 2000).

### Comparison between DNA damage levels of B and T lymphocytes

Comparisons between DNA damage levels of B and T lymphocytes showed that the levels of damage were correlated in these two subpopulations of lymphocytes (*r* = 0.69 for the correlation of both groups of individuals (non-exposed and FO) at the three time points (samples S0, S2 and S4) (for detailed results, refer to Figure S2). The correlation was slightly higher (*r* = 0.73) in the non-exposed group than in the FO group (*r* = 0.66). To decipher this point, B and T lymphocyte DNA damage was compared among the FO group separately in the S0, S2 and S4 samples. The highest correlation was observed at the time point corresponding to sample S0 (*r* = 0.80). These results suggest that the spraying season does not always induce the same variation in DNA damage levels between B and T lymphocytes, thus explaining the weaker correlation between the two subgroups of lymphocytes during the spraying season.

### Cross-sectional analyses

#### Cross-sectional analysis in farm owners’ and non-exposed individuals’ samples

Viability and lymphocyte DNA damage, as determined by trypan blue exclusion method and comet assays on B or T lymphocytes, respectively, are presented for both groups (Supplementary Table S1 and Supplementary Table S2, respectively). The OTM parameters obtained from the standard used in each experiment are also reported in Supplementary Tables S1 and S2. No significant association was observed between DNA damage in P0 (relative median OTM) and age for both groups and type of lymphocytes (*ρ* = 0.09, *P* = 0.68 for B lymphocytes in the non-exposed group; *ρ* = −0.15, *P* = 0.52 for T lymphocytes in the non-exposed group; *ρ* = −0.02, *P* = 0.93 for B lymphocytes in the FO group; *ρ* = 0.74, *P* = 0.08 for T lymphocytes in the FO group). Among potential confounding factors, no significant association was observed between DNA damage and current smoking status (Wilcoxon’s test, *P* = 0.41 for B lymphocytes and *P* = 0.78 for T lymphocytes) nor with alcohol consumption (Wilcoxon’s test, *P* = 0.25 for B lymphocytes and *P* = 0.21 for T lymphocytes), even if a slightly decreased level of DNA damage (25 and 30 % for B and T lymphocytes, respectively) was observed for daily drinkers with a borderline significant negative correlation with the quantity of alcohol expressed in g per day for T lymphocytes (*P* = 0.06, *ρ* = −0.46). Because sunlight could contribute to the variations in DNA damage level observed between the two periods, we examined its influence by collecting daily sunlight data from the nearest meteorological station to each farm. No significant correlation was observed between DNA damage and sunlight radiation as estimated by using the individual cumulative sunlight record the week before sampling (*P* = 0.81; *ρ* = 0.06 and *P* = 0.34; *ρ* = −0.22 for B lymphocytes and T lymphocytes, respectively).

#### Cross-sectional analysis between farm owners and non-exposed individuals and association of DNA damage with farm-related activities

The relative median OTMs in both groups were similar in P0 for B lymphocytes (*P* = 0.12) and T lymphocytes (*P* = 0.63). However, median OTMs of B lymphocytes showed a trend to be slightly higher in the FO group than in the non-exposed group during P0 (relative median OTM = 1.53 in the FO group versus 1.30 in the non-exposed group, *P* = 0.12). In P4, the relative median OTMs were significantly higher in the FO group than in the unexposed group (*P* = 0.003 for B lymphocytes and *P* = 0.001 for T lymphocytes).

Characteristics of farm-related activities are presented in Table 2. All FO grew wheat, most of them had peas and sugar beet, half of them grew corn, and a minority was also fruit growers. Interestingly, FO growing sugar beet had significantly higher levels of T lymphocyte DNA damage in both P0 (*P* = 0.01) and P4 (*P* = 0.04). Fifty percent of FO also had livestock; six were cattle farmers including two dairy farmers, one raised sheep and one owned a poultry farm. Livestock farming did not significantly influence DNA damage. In addition, no correlation was observed between total farm area or farm area devoted to crops and median OTM in P0 and P4. FO had used pesticides on crops for 17 years on average, but there was no correlation with DNA damage.

### Longitudinal analyses

#### Seasonal effects in farm owners and non-exposed individuals

**Fig. 2:**
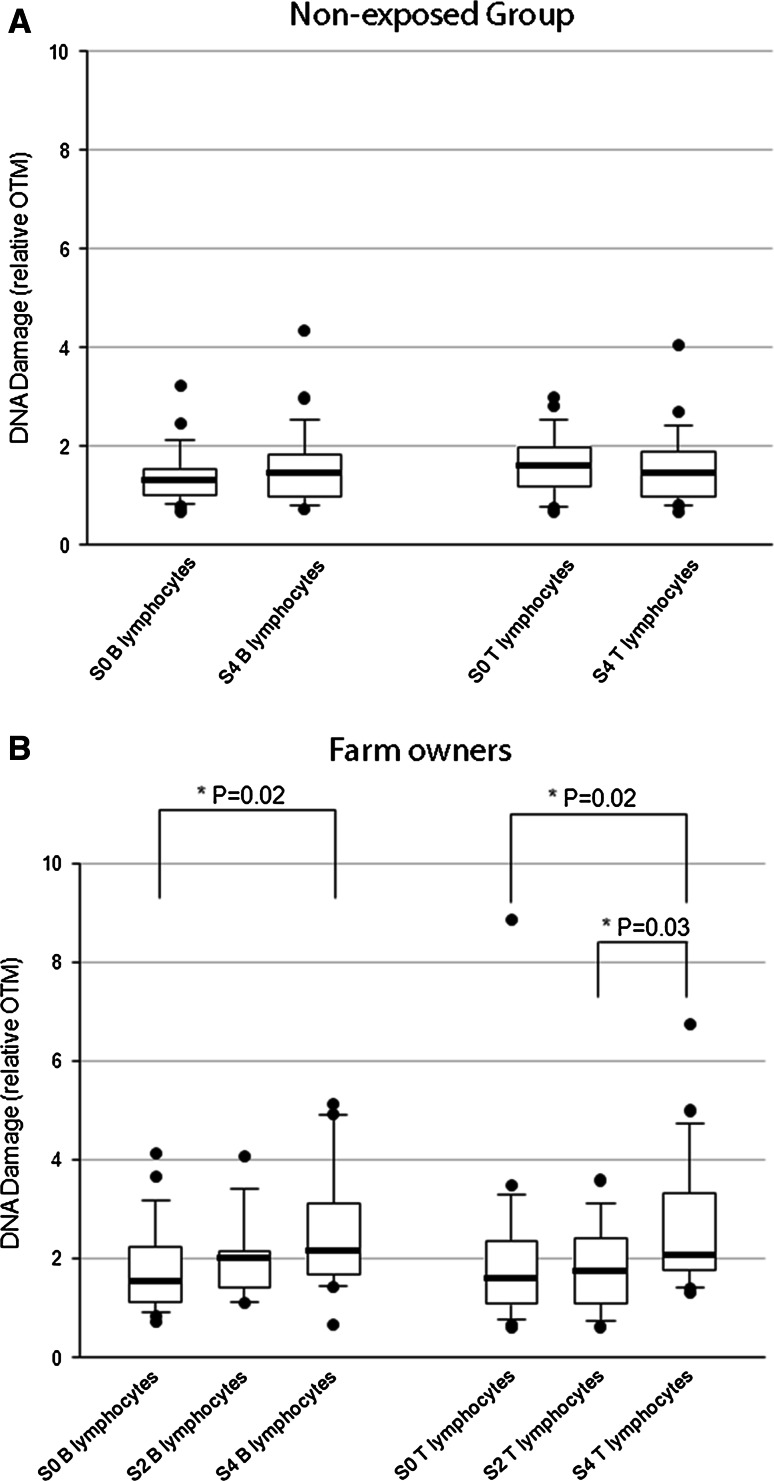
DNA damage on B and T lymphocytes in non-exposed group (**a**) and farm owners group (**b**). For both groups, S0 samples were collected at P0 period (January) and S4 samples were collected at period corresponding to the intensive spraying period P4 (May–June). For the farm owners group, another sample (S2 sample) was collected at P2 period (after the first day of spraying season). *Boxes* are limited by first and third quartiles separated by the median; thin *vertical lines* represent minimum and maximum values in the 10–90th percentile range. Points are the extreme values. Statistical analysis was performed with paired samples Student’s *t* test

Various parameters [minimum, percentiles (25, 50, 75) and maximum] were calculated on relative OTMs, and the results are presented as box plots in Fig. 2a, b. Figure 2a shows DNA damage level in the non-exposed group for both B and T lymphocyte in P0 period (January) and P4 (May–June). Figure 2b shows DNA damage level in the FO group for both B and T lymphocytes in P0 (January), P2 (after the second day of pesticide spraying) and P4 (May–June).

Differences in median OTMs of B (or T lymphocytes) between P4 and P0 were not statistically significant in the non-exposed group (*P* = 0.31 for B lymphocytes and *P* = 0.72 for T lymphocytes) (Fig. 2a). Statistical analysis revealed similar results when three unexposed volunteers, for whom the OTM of the standard used in the experiment was high (OTM > 0.4, see Fig. 1), were excluded from the analysis. In FO, the difference between OTMs of B and T lymphocytes was statistically significant between P0 and P4 (*P* = 0.02 for B lymphocytes and *P* = 0.02 for T lymphocytes), showing a seasonal effect. The median OTM of B and T lymphocytes in the FO group was higher during the spraying period (P4) than before (P0) (Fig. 2b). Relative median OTMs were also evaluated for P0, P2 and P4 in order to compare DNA damage between P0 and P2 and between P2 and P4. No statistically significant differences were observed between P0 and P2 either for B lymphocytes (relative median OTM = 1.53 at P0 vs. 2.00 at P2) or for T lymphocytes (relative median OTM = 1.58 at P0 vs. 1.75 at P2). DNA damage was significantly higher (*P* = 0.03) in P4 than in P2 with regard to T lymphocytes (relative median OTM = 2.06 at P4 vs. 1.75 at P2). In contrast, there was no significant difference (*P* = 0.17) between OTMs in P2 and P4 for B lymphocytes (relative median OTM = 2.16 at P4 vs. 2.00 at P2).

Seasonal effects were also significant with regard to total human mononuclear cells in FO (*P* = 0.0001) but not in the non-exposed group (*P* = 0.47).

#### Correlation between seasonal effect and farmers’ exposure characteristics

Among pesticides, herbicides were the most frequently used (average of 12 days of use between the two samples collected in P0 and P4) and followed by fungicides and insecticides (respectively, used for an average of 6 and 3 days of treatments during the same period). The chemical classes of pesticides used have been previously described (Le Goff et al. 2005). The pesticides handled during a whole season of treatment covered a wide range of molecules (136 active ingredients) from 60 chemical classes. FO used pesticides 21 days on average between P0 and P4. Interestingly, there was a tendency showing a potential effect of the number of days of fungicide treatments (*r* ^2^ = 0.43; *P* = 0.11) between the two time points with regard to T lymphocyte DNA damage but no trend for herbicide (*r* ^2^ = 0.24; *P* = 0.39) or insecticide (*r* ^2^ = 0.14; *P* = 0.63) use. Between S0 and S4 samples, FO used 12 chemical families of fungicides including 23 different active ingredients. Almost all of the FO used at least one active ingredient among the triazole fungicides and more than 50 % used three other chemical families (i.e., anilinopyrimidine, piperidine or chlorothalonil) as described in Table 3.

**Table 3:** Description of chemical families of fungicides used on crops between the S0 and S4 samples, with classifications according to CLP regulations and IARC monographies

Chemical family (*active ingredients*)	% use
Triazole (*cyproconazole* ^a^, *epoxiconazole* ^a,b^, *fluquinconazole*, *flutriafol*, *hexaconazole*, *metconazole* ^a^, *tebuconazole* ^a^, *tetraconazole*)	93
Anilinopyrimidine (*cyprodinil*)	73
Piperidine (*fenpropidin*)	60
Benzene derivatives (*chlorothalonil* ^b,f^)	53
Dithiocarbamates (*maneb* ^a/g^, *mancozeb* ^a^, *thiram* ^g^)	47
Benzimidazoles (*carbendazim* ^c,d^, *thiophanate* *methyl* ^e^)	40
Acetamide (*cymoxanil*)	33
Phenylamides (*oxadixyl*)	27
Morpholines (*fenpropimorph* ^a^, *tridemorph* ^d^)	20
Phenylpyridylamine (*fluazinam*), formamide (*triforine*), phenylurea (*pencycuron*)	7

*CLP* classification, labeling and packaging, *IARC* International Agency for Research on Cancer

^a^Classified R2(H361d), ^b^classified C2, ^c^classified M1B, ^d^classified R1B(H360d), ^e^classified M2, according to the European Union CLP regulation no. 1272/2008, ^f^classified 2B and ^g^classified 3, according to the IARC monographies (1987, 1991, 1999)

With regard to regulation labeling in the European Union concerning these fungicides, chlorothalonil and benzimidazoles (carbendazim and thiophanate methyl) are suspected to be carcinogenic or mutagenic. The mean difference between OTMs at P0 and P4 for the T lymphocytes in FO who used at least one of these three fungicides was +0.16 versus −0.01 for FO who used other fungicides (paired *t* test, *P* = 0.15). The influence of the type of pesticide spraying materials was not evaluated since almost all FO (16 out of 20) used the same type of sprayer (a trailer sprayer with a tank volume ranging from 2,000 to 3,200 L and a boom length of 18 to 28 m). Furthermore, the potential protective effect of personal protective equipment could not be studied since no farmer used any.

Results of longitudinal and cross-sectional analyses detailed in the manuscript are summarized in the supplementary material (Supplementary Figure S1).

## Discussion

In this biomonitoring study using comet assays, we observed a statistically significant genotoxic effect of the pesticide spraying season in both B (*P* = 0.02) and T lymphocytes (*P* = 0.02) from farm owners. In contrast, non-exposed individuals did not show any significant modifications.

Although age, gender and smoking are factors commonly thought to influence the level of DNA damage measured by the comet assay, conflicting reports have been published (Faust et al. 2004; Moller et al. 2000, 2002). A review of 125 biomonitoring studies (Moller 2006) concluded that there was no effect of gender and smoking status on DNA damage level measured on blood cells, measured by Comet assay. The influence of gender was not an issue in our study as we included only males. No influence of smoking status was detected in our groups. We did not observe any effect of age on DNA damage, although the age range was narrow (25–52 years). Our results are in accordance with the majority of studies reporting no effect of age, although a few studies have reported a positive association of age on DNA damage, mainly when people older than 55 or 60 years old were compared with younger people (Faust et al. 2004; Moller 2006; Moller et al. 2000). Excessive exercise may also be an important confounding factor for the interpretation of comet assay data. In our study, all blood samples were obtained in the same conditions and at rest in the morning before any job activity in order to reduce the effect of physical exercise (Moller et al. 2000).

As described, we took into account various confounding factors in this study. Moreover, most of our statistic analyses were based on longitudinal comparisons as different samples from the same volunteer were collected. Such a longitudinal design where each volunteer served as his own control greatly reduced the influence of most of the individual confounding factors (Bull et al. 2006; Moller et al. 2000). In this biomonitoring study, FO were exposed chronically to multiple pesticides at different levels. Such a complex form of exposure can induce many types of DNA damage detectable by the alkaline comet assay such as single-strand breaks, alkali-labile sites and double-strand breaks. Such DNA damage is repaired by various mechanisms including base excision repair for oxidative damage and single-strand breaks and the non-homologous end-joining pathway for double-strand breaks (Parsons and Dianov 2013; Wang and Lees-Miller 2013). These DNA repair pathways are likely to be differentially efficient in B and T cells. The mechanisms underlining these disparities have not been completely elucidated although differences in DNA repair capacity have been suggested. In addition, the nature of pesticides and level of exposure may have varied among the individuals exposed in our study. Altogether, these factors could explain why in some subjects B lymphocytes displayed a higher, similar or lower level of DNA damage than T lymphocytes. Differences in the genetic backgrounds (e.g., on DNA repair enzymes or xenobiotic-metabolizing enzymes) of subjects could also influence DNA damage and repair capacity after such complex exposure. Additional studies are thus needed in order to elucidate the differential sensitivity of B and T lymphocytes to genotoxic agents after either acute or chronic exposure.

The present findings confirm the ability of occupational pesticide exposure to increase levels of genotoxic damage, consistently with previous reports (reviewed in Bolognesi 2003; Bull et al. 2006). Lymphocytes from FO displayed a higher amount of DNA damage in samples collected during the spraying season (P4, May–June) than in those collected before it (P0, January). Various studies have reported seasonal variations in DNA damage in control subjects, mainly in July and August, compared to other periods of the year (Giovannelli et al. 2006; Moller et al. 2000, 2002). Such variations were mainly attributed to an increase in solar exposure during the summer months. Increased levels of DNA damage in mononuclear cells may be explained by the penetration of sunlight into the epidermal outer layer in humans. This might cause DNA damage to peripheral mononuclear cells circulating in the vessels of the skin (Moller et al. 2002). In addition, seasonal variations in DNA damage may be due to changes in air pollution levels (Giovannelli et al. 2006). The influence of these two factors is preponderant during July and August in France. In our study, samples were collected during winter and spring, seasons when the influence of sunlight and pollution cofactors is usually lesser (Giovannelli et al. 2006; Moller et al. 2000, 2002), and we found no seasonal effect in non-exposed subjects. Moreover, no correlation was observed between levels of DNA damage and solar irradiation during the week preceding sampling.

Our findings provide some evidence that the changes observed in DNA damage were due to pesticide exposure. Since workers are usually exposed to complex mixtures of pesticides, it is difficult to attribute genotoxic effects to any particular chemical class or compound. However, fungicides may have played a key role in inducing DNA damage on T lymphocytes. We observed a trend between changes in the level of DNA damage and the number of days of fungicide treatments between the S0 and S4 samples. Adding each of the potential confounding variables (age, smoking, alcohol consumption or UV exposure) in a multiple regression model did not change the level of suggested effect of the number of days of fungicide use (data not shown). Moreover, the increase in the level of DNA damage was limited to the 15 FO who used at least one of the three fungicides suspected of being carcinogenic or mutagenic: chlorothalonil, carbendazim and thiophanate methyl. In a previous study among FO using chlorothalonil in combination with insecticides, we observed a significant increased level of DNA damage after exposure (Lebailly et al. 1998b). In our previous in vitro study using comet assays on CHO-K1 cells, genotoxic effects of chlorothalonil were also observed (Vigreux et al. 1998). Moreover, chlorothalonil, which was initially classified in category 3 by the IARC in 1991 [International Agency for Research on Cancer (IARC) 1987, 1991], was reclassified in category 2B in 1999 (1999). In vivo studies on rodents subchronically exposed to pesticides (28 days of exposure) demonstrated that carbendazim in combination with imazalil, another fungicide, potentiated the ability of the latter to induce DNA damage (Ethikic et al. 2012). In a critical review of the literature, Bull et al. 2006 showed that among the 24 good-quality studies, 71 % demonstrated an effect of pesticide use. Several pesticides were evaluated in these studies, and fungicides were often used. The three fungicides identified in the present study were assessed in six other studies: Four of them demonstrated significant changes in DNA adducts (Peluso et al. 1996), chromosomal aberration (De Ferrari et al. 1991; Lander et al. 2000) or comet assay (Grover et al. 2003) but not the two others on micronucleus (Bolognesi et al. 2004; Falck et al. 1999). Our results based on a genotoxicity biomarker suggest that occupational pesticide exposure induces cumulative effects, as previously indicated by the majority of studies based on cytogenetic biomarkers (chromosomal aberrations, micronuclei or sister chromatid exchange) (for review, see Bolognesi 2003).

Further detailed evaluation of pesticide genotoxicity is thus required. Individuals occupationally exposed to various mixtures of pesticides should be biomonitored in order to detect genotoxic effects early and prevent the induction of DNA damage, which could be the initial step of carcinogenesis. Moreover, results on cancer incidence from the Agricultural Health Study Cohort have demonstrated consistent relations with exposure mainly to insecticides and herbicides (for review, see Weichenthal et al. 2010). However, in the Agricultural Health Study Cohort, very few people were exposed to fungicides. Further epidemiologic studies are required to evaluate the potential carcinogenicity of pesticides and especially fungicides.

## Electronic supplementary material

Below is the link to the electronic supplementary material.

Supplementary material 1 (DOC 537 kb)

## Abbreviations

CLP: Classification, labeling and packaging
EFS: *Etablissement Français du Sang*
FO: Farm owners
IARC: International Agency for Research on Cancer
OTM: Olive tail moment
PBL: Peripheral blood lymphocytes
SD: Standard deviation
