# Supplementary material for: DNA damage in B and T lymphocytes of farmers during one pesticide spraying season
Source: Int Arch Occup Environ Health. 2015 Feb 3;88(7):963–72. doi: 10.1007/s00420-015-1024-3 (PMC4564440; doi:10.1007/s00420-015-1024-3)

# Supplemental Material

## DNA damage in B- and T-lymphocytes of farmers during one pesticide spraying season

Pierre Lebailly, Gladys Mirey, Fabrice Herin, Yannick Lecluse, Bernard Salles and Elisa Boutet-Robinet

**Table S1. Viability and DNA damage of cells from volunteers in** the non-exposed group

| Volunteer n° | % of viability | | Median OTM | | | | |
| --- | --- | --- | --- | --- | --- | --- | --- |
|  | Unsorted cells of volunteer | | Internal standard | B-lymphocytes | | T-lymphocytes | |
|  | Period | |  | Period | | Period | |
|  | January (S0) | May-June (S4) | for samples S0 and S4 | January (S0) | May-June (S4) | January (S0) | May-June (S4) |
| 91 | 82% | 89% | 0.22 | 0.37 | 0.16 | 0.44 | 0.19 |
| 93 | 91% | 85% | 0.16 | 0.15 | 0.28 | 0.18 | 0.28 |
| 95 | 87% | 92% | 0.20 | 0.40 | 0.33 | 0.57 | 0.54 |
| 96 | 87% | 95% | 0.21 | 0.25 | 0.20 | 0.29 | 0.26 |
| 99 | 94% | 94% | 0.30 | 0.37 | 0.46 | 0.52 | 0.59 |
| 100 | 92% | 94% | 0.20 | 0.49 | 0.26 | 0.48 | 0.29 |
| 101 | 93% | 88% | 0.19 | 0.34 | 0.37 | 0.26 | 0.36 |
| 102 | 90% | 86% | 0.22 | 0.29 | 0.41 | 0.35 | 0.32 |
| 103 | 97% | 87% | 0.30 | 0.21 | 0.41 | 0.21 | 0.29 |
| 109 | 89% | 88% | 0.16 | 0.13 | 0.17 | 0.23 | 0.23 |
| 110 | 91% | 88% | 0.60 | 0.89 | 0.48 | 1.06 | 0.48 |
| 112 | 93% | 92% | 0.35 | 0.45 | 0.28 | 0.28 | 0.29 |
| 114 | 94% | 93% | 0.30 | 0.43 | 0.64 | 0.90 | 0.27 |
| 115 | 91% | 83% | 0.46 | 0.46 | 1.09 | 0.76 | 1.05 |
| 117 | 90% | 91% | 0.48 | 0.47 | 0.88 | 0.36 | 0.86 |
| 118 | 94% | 87% | 0.27 | 0.42 | 0.42 | 0.44 | 0.49 |
| 119 | 95% | 83% | 0.20 | 0.65 | 0.87 | 0.44 | 0.81 |
| 120 | 95% | 96% | 0.28 | 0.37 | 0.29 | 0.33 | 0.19 |
| 124 | 93% | 91% | 0.18 | 0.15 | 0.54 | 0.39 | 0.40 |
| 125 | 97% | 95% | 0.21 | 0.21 | 0.19 | 0.24 | 0.24 |
| 126 | 91% | 92% | 0.23 | 0.22 | 0.21 | 0.30 | 0.24 |
| 127 | 95% | 93% | 0.20 | 0.27 | 0.19 | 0.32 | 0.23 |

**Table S2. Viability and DNA damage of cells from volunteers in the farm owners** group

| Volunteer n° | % of viability | | | | Median OTM | | | | | | | | | |
| --- | --- | --- | --- | --- | --- | --- | --- | --- | --- | --- | --- | --- | --- | --- |
|  | Unsorted cells of volunteers | | | | Internal standard | | B-lymphocytes | | | | T-lymphocytes | | | |
|  | Period | | | |  | | Period | | | | Period | | | |
|  | S0 | S2 | S3 | S4 | for samples S0 and S4 | for samples S2 and S3 | S0 | S2 | S3 | S4 | S0 | S2 | S3 | S4 |
| 60 | 97% | 97% |  | 97% | 0.10 | 0.17 | 0.16 | 0.35 |  | 0.21 | 0.16 | 0.61 |  | 0.34 |
| 62 | 99% | 97% | 98% | 98% | 0.23 | 0.17 | 0.25 | 0.38 | 0.22 | 0.39 | 0.15 | 0.19 | 0.25 | 0.47 |
| 63 | 88% |  |  | 96% | 0.23 |  | 0.84 |  |  | 0.34 | 0.80 |  |  | 0.44 |
| 65 | 93% |  |  | 92% | 0.30 |  | 0.44 |  |  | 0.43 | 0.33 |  |  | 0.43 |
| 66 | 98% |  |  | 98% | 0.19 |  | 0.16 |  |  | 0.30 | 0.27 |  |  | 0.38 |
| 67 | 98% | 98% |  | 98% | 0.10 | 0.16 | 0.19 | 0.28 |  | 0.44 | 0.20 | 0.34 |  | 0.50 |
| 69 | 91% |  |  | 92% | 0.33 |  | 0.24 |  |  | 0.54 | 0.20 |  |  | 0.64 |
| 70 | 93% | 95% | 94% | 94% | 0.18 | 0.18 | 0.30 | 0.20 | 0.39 | 0.45 | 0.28 | 0.14 | 0.42 | 0.60 |
| 74 | 96% | 98% |  | 96% | 0.08 | 0.16 | 0.33 | 0.33 |  | 0.39 | 0.71 | 0.36 |  | 0.54 |
| 75 | 95% | 98% |  | 96% | 0.14 | 0.15 | 0.14 | 0.61 |  | 0.31 | 0.34 | 0.44 |  | 0.29 |
| 76 | 90% | 97% |  | 81% | 0.22 | 0.15 | 0.32 | 0.32 |  | 0.46 | 0.39 | 0.45 |  | 0.61 |
| 77 | 91% | 96% |  | 92% | 0.08 | 0.21 | 0.16 | 0.36 |  | 0.41 | 0.25 | 0.39 |  | 0.18 |
| 78 | 90% | 86% |  | 93% | 0.18 | 0.12 | 0.49 | 0.39 |  | 0.34 | 0.42 | 0.21 |  | 0.34 |
| 80 | 98% | 97% | 96% | 99% | 0.16 | 0.14 | 0.40 | 0.28 | 0.30 | 0.48 | 0.18 | 0.14 | 0.13 | 0.21 |
| 83 | 91% | 96% |  | 89% | 0.22 | 0.21 | 0.40 | 0.29 |  | 0.45 | 0.44 | 0.13 |  | 0.36 |
| 84 | 97% | 83% |  | 98% | 0.18 | 0.12 | 0.22 | 0.17 |  | 0.12 | 0.19 | 0.21 |  | 0.25 |
| 85 | 97% |  |  | 98% | 0.12 |  | 0.14 |  |  | 0.39 | 0.13 |  |  | 0.38 |
| 86 | 98% | 98% |  | 97% | 0.11 | 0.17 | 0.29 | 0.19 |  | 0.25 | 0.31 | 0.29 |  | 0.49 |
| 88 | 98% |  |  | 97% | 0.15 |  | 0.15 |  |  | 0.74 | 0.13 |  |  | 0.50 |
| 90 | 95% |  |  | 92% | 0.30 |  | 0.34 |  |  | 0.71 | 0.28 |  |  | 0.46 |

**Figure S1. Summary of longitudinal and cross-sectional statistical analyses. For each sample (S0, S2 and S4), relative median OTM is presented for both B-lymphocytes (Bλ) and T-lymphocytes (Tλ).**

**
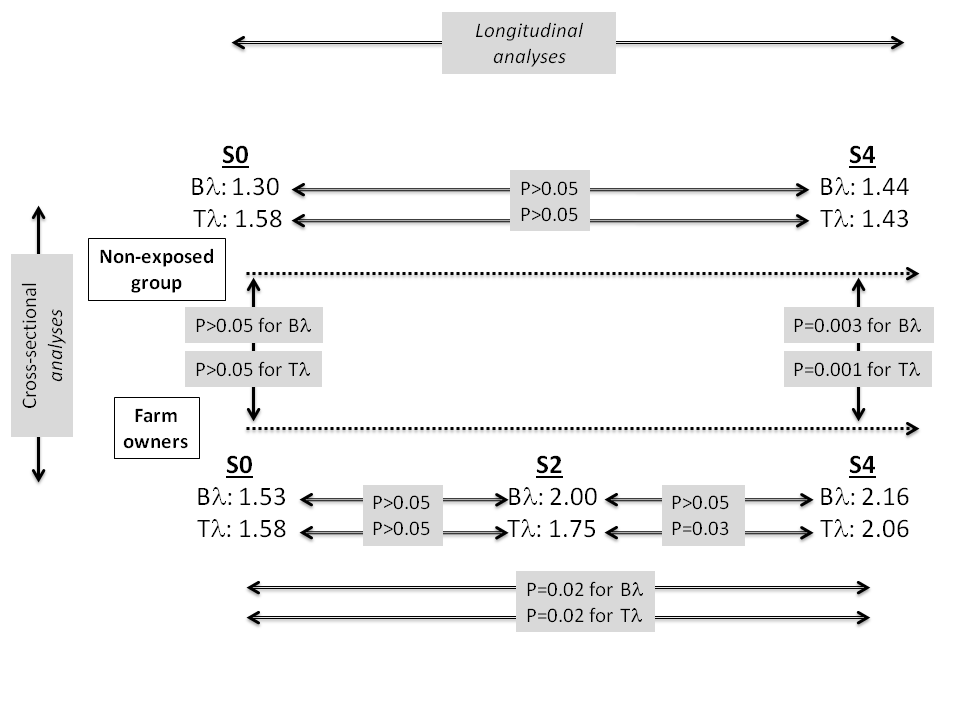
**

**Figure S2. Correlation between B- and T- lymphocytes DNA damage levels.**

Each solid line represents the linear regression line of each data set. Each dotted line represents the line with the equation Y=X. For each data set, the equation of the linear regression line and the Pearson correlation coefficient (r) are detailed under the graph.

(a) Relative median Olive Tail Moment (OTM) of B- and T-lymphocytes from both groups (farm owners and non-exposed groups) for samples S0, S2, S4 corresponding to the different time points. (b) Relative median OTM of B- and T-lymphocytes from the non-exposed group for the samples S0 and S4. (c) Relative median OTM of B- and T-lymphocytes from the farm owners group for samples S0, S2 and S4. (d) Relative median OTM of B- and T-lymphocytes from the farm owners group for samples S0. (e) Relative median OTM of B- and T-lymphocytes from the farm owners group for samples S2. (f) Relative median OTM of B- and T-lymphocytes from the farm owners group for samples S4.


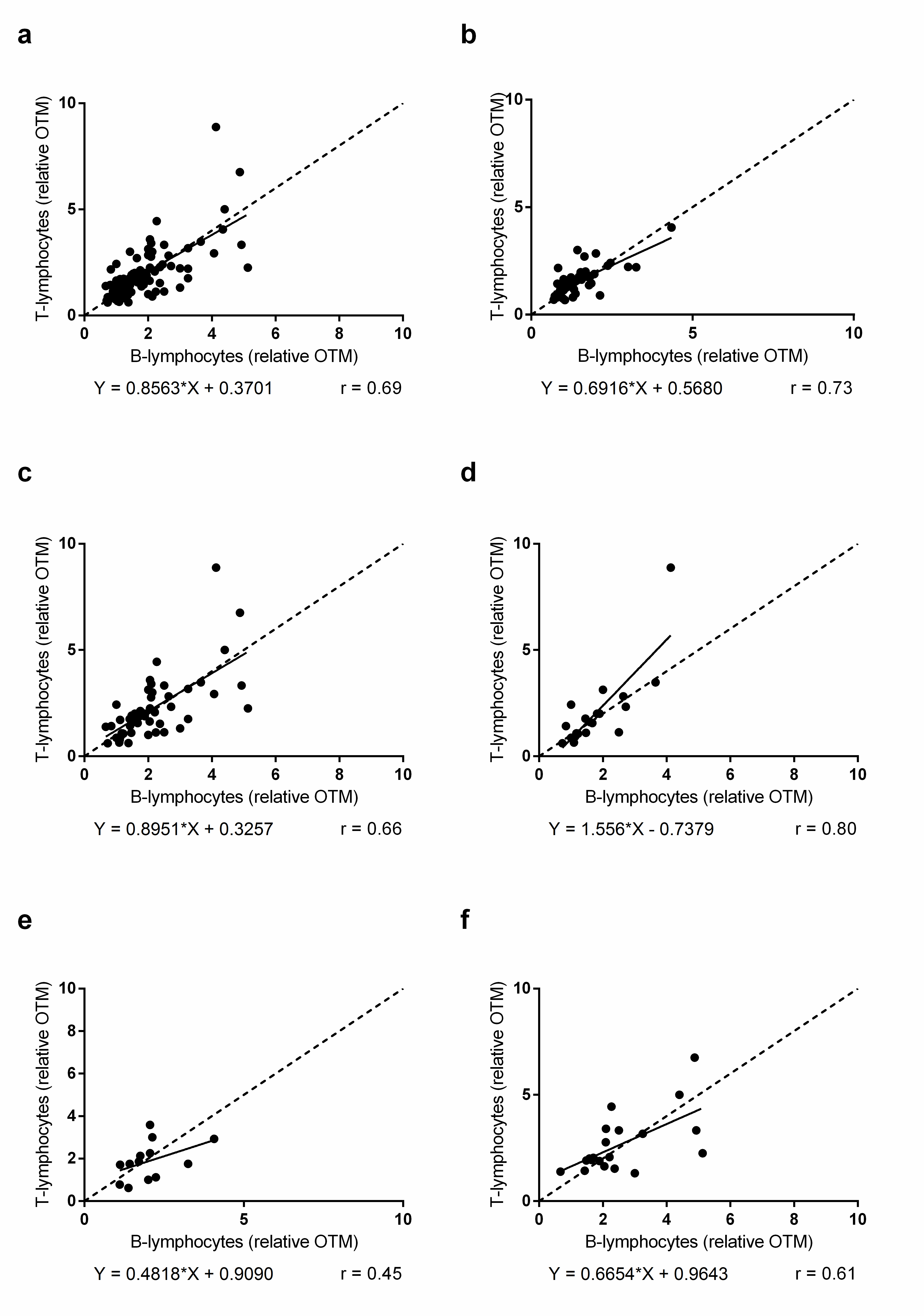

Supplement: Supplementary file 1 — Supplementary material 1 (DOC 537 kb) [file 420_2015_1024_MOESM1_ESM.doc]
